# Trustworthy agent-based simulation: the case for domain-specific modelling languages

**DOI:** 10.1007/s10270-023-01082-9

**Published:** 2023-02-08

**Authors:** Steffen Zschaler, Fiona A. C. Polack

**Affiliations:** 1 Department of Informatics, King’s College London, London, UK; 2 School of Computer Science, University of Hull, Hull, UK

## Abstract

Simulation is a key tool for researching complex system behaviour. Agent-based simulation has been applied across domains, such as biology, health, economics and urban sciences. However, engineering robust, efficient, maintainable, and reliable agent-based simulations is challenging. We present a vision for engineering agent simulations comprising a family of domain-specific modelling languages (DSMLs) that integrates core software engineering, validation and simulation experimentation. We relate the vision to examples of principled simulation, to show how the DSMLs would improve robustness, efficiency, and maintainability of simulations. Focusing on how to demonstrate the fitness for purpose of a simulator, the envisaged approach supports bi-directional transparency and traceability between the original domain understanding to the implementation, interpretation of results and evaluation of hypotheses.

## Introduction

Complexity is inherent to life; most areas of science and policy benefit from an understanding of complex systems. It is a feature of complexity that instrumenting a system in order to experiment on it directly disrupts the natural patterns of interaction [9, 15, 27]: experimental results are at best approximate. There are also significant ethical problems with experimentation: it is ethically undesirable to use animal models, common in medical and biological research, because the experimental set-up disrupts the systems under study and harms (often kills) the experimental subjects. Furthermore, experimentation on live, or recently dead, organisms is not strictly repeatable or reproducible, as the organism and the environment are unique and complex. In a similar way, experimenting on human and engineered complex systems is disruptive and potentially unethical: we cannot experiment safely on economic and social systems (though politicians like to try), or on complex safety-critical systems such as aircraft or chemical/nuclear plant controllers.

Simulation offers a computational alternative to live experiments. If a complex system behaviour can be suitably modelled, then repeatable and reproducible experiments can be run, limited only by computational resources. For a simulation to be trusted, it must be demonstrable that simulation observations are the outcome of appropriately captured behaviours, not experiment artefacts or coding errors.

An example of the trust problem can be seen in recent high-profile agent-based and mathematical simulations supporting research on the COVID-19 pandemic. The simulation designs and code have attracted substantial criticism from the software-engineering community, not least for the lack of recorded rationale (notably parameter value selection). Scientists have declared their confidence that their simulations are sufficient analogues of reality, but this does not amount to demonstrable fitness for purpose, and makes it difficult to challenge and improve the models. Furthermore, the simulations are hard to develop further, reliant on the knowledge and skill of the original developers.

### Principled simulation

**Fig. 1:**
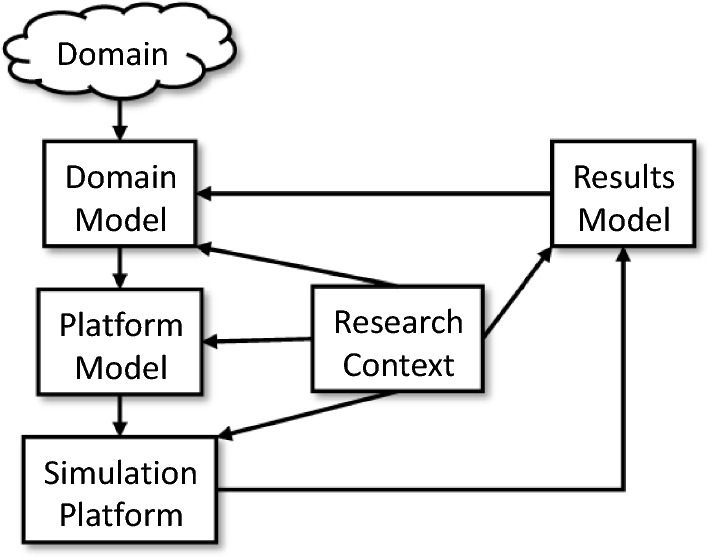
Overview of the CoSMoS approach [44]

Although there are partial solutions to simulation engineering (cf. Sect. 6), most neglect the important aspect of fitness-for-purpose, and its connotations for *principled* complex-systems simulation. The importance of establishing and recording aspects that lead users or developers to trust the engineering and the resultant simulation has been captured in, for example, the CoSMoS Process [44]. CoSMoS is a ”lifecyle model” (see Fig. 1) of the whole simulation process, from domain exploration and identification of an appropriate simulation focus, to interpretation of results. CoSMoS is taken as the context for our vision of simulation engineering.

No techniques or work flows are mandated by CoSMoS, but in projects that have used the approach the modelling has been informal—often using *ad hoc* variants of UML. As a result, whilst developers have used programming environments, none has taken up CoSMoS’s suggestion of model-driven engineering. Some of the problems resulting from the lack of a coherent development approach are:

- simulation development that follows appropriate modelling, but is coded manually, which is error prone, difficult to trace, and hard to support with a robust argument of fitness;
- experimentation built incrementally into the simulation codebase, making it difficult to modify the simulator or the experiments—and making it difficult to demonstrate (to outsiders) how the *in silico* experiments relate to real-world experimentation and observation;
- experiments designed directly in code, a notation that is usually unfamiliar to domain experts, reducing understanding, and thus trust, of what the simulation experiment represents cf. the real world, as well as limiting potential for identifying new experiments.
- simulations that are fine so long as the original experts and engineers are using them, but are not maintainable, extendable or re-usable by others, resulting in many wasted hours of development, and failure of the fitness arguments (loss of understanding of the basis for trusting the simulation and its results).

These issues mean that simulation development continues to be a specialist activity, non-viable for many complex scenarios that would benefit substantially from computer simulation. Our vision is of well-founded, tool-supported notations, supporting CoSMoS-like development that encompass domain and scope exploration, software design and implementation, as well as experimental design and the recording of rationale. The later aspect, for which CoSMoS recommends argumentation techniques needs underpinning with traceability to development and experimentation designs. Our vision also needs to support the interpretation of simulation results into their real-world context, again through linkages across the family of models facilitating model management.

The vision underpins principled simulation with automation, with the potential to remove many of these problems and opportunities for error from the simulation development workflow. The envisaged basis for automation is a family of DSMLs to express features of the domain and simulation, as well as queries on the development. A DSML approach would allow domain experts to directly interact with simulation models and experiment specifications, while supporting the automatic translation of models and specifications into executable simulations.

Crucially, our envisaged family of DSMLs includes languages for expressing expected behaviours and fit-for-purpose arguments, integrated at a fundamental level with executable simulations, as well as languages for specifying appropriate simulation experiments and experimental protocols, with appropriate validation and sensitivity analysis to allow robust conclusions to be drawn. Based on our experience, the vision is expressed for agent-based simulations, but we believe that it would generalise to other forms of simulations.

This paper is an extension of [52], adding the following contributions:

1. A detailed discussion of four user scenarios where a model-driven approach to simulation development can be beneficial, including prototypical application to the motivating scenario from [1, 2]; and
2. A broader and more in-depth discussion of related work.

In the remainder of this paper, we first present a motivating example and highlight some challenges (Sect. 2). Section 3 presents an overview of our vision for a family of DSMLs for agent-based simulation. In Sect. 4 we introduce four user scenarios, which we discuss in detail in Sect. 5 applying them to the challenges identified in Sect. 2. Finally, we discuss other efforts for systematic engineering of agent-based simulations in Sect. 6 and conclude in Sect. 7.

## Motivating example

The motivating example is hypothetical but based on simulator development to support laboratory work at York Computational Immunology Laboratory on (a) formation of Peyer’s Patch cell clusters [1, 2];^1^ and (b) granuloma formation in visceral Lieshmaniasis [33]. For each project, an experienced laboratory team worked with CoSMoS team members from engineering disciplines. Four PhDs (and some other PhD and student projects), investigated different hypotheses; models and code were developed, exchanged, extended, and abandoned. The series of simulator developments, following the CoSMoS principles, were implemented on Java Mason, Repast and Flame agent platforms [17, 26, 33, 49].

The PPSim (Peyer’s Patch Simulator) was developed to investigate laboratory research hypotheses relating to cell cluster development in a neonatal mouse gut. The simulator development involved modelling the domain (cells, chemicals and interactions), validating models with the laboratory scientists, software modelling, and implementation on an agent-based platform, developing bespoke code for component behaviours. The rationale for the development is carefully documented, with argumentation diagrams and extensive text recording the belief in the fitness for purpose of the simulator. Experimentation *in vivo* and *in silico* established new understanding of the triggers to cluster formation (see [1, 2, 4]). The collaboration lasted some 6 years (2010–2017). The simulator was available as a downloadable application until 2021, latterly at the Kennedy lab, in Oxford,^2^ and included manually-developed guidance and scripts for rerunning existing experiments, and for calibration and sensitivity analysis.

In the hypothetical analogy, as in reality, having developed the original PPSim application, the software engineer (SE1) moved away. However, the team now wants to study cluster distribution, and also to use simulation to explore other cell cluster formation. This needs modification of simulation components and behaviours. The team has the ability to modify the domain-modelling for the new hypotheses, but not the expertise to modify the implementation. It must also keep the recording and the fitness for purpose argumentation up to date, and ensure that existing experiments still run.

A new software engineer (SE2) joins the team. To understand the design, implementation, rationale and fitness-for-purpose arguments, SE2 needs to recreate all SE1’s knowledge acquisition. Technical problems arise: missing version histories, historical incompatibility of open-source platforms or code. Inevitably, the simulator documentation does not provide all the detail needed to understand the code: it is clear what the components are, and how they are intended to be implemented, but not how each agent and interaction is coded on the Java Mason platform.

Subsequently, another project wants to adapt the simulation to explore a “granuloma formation” hypothesis. SE2 starts to map out how to replace PPSim cells with granuloma-forming cells. There are 1:1 mappings between the implicated cell types, but some interaction behaviour and timing parameters (and the cell environment) are not identical. SE2 has solved some of the technical issues, but the code, which comprises the platform representation of agents along with the bespoke coding of interaction triggers and behaviours, bespoke agent environment, and bespoke visualisations and data capture—created by two independent developers with different coding styles—defies systematic modification.

*Key challenges* arising in this example include:

- The lack of formal mapping between designs and code makes traceability subjective. When design models change, code cannot be simply regenerated.
- The agent platform’s agent architecture distributes code for an agent across classes, making it difficult to relate one cell agent to a coherent block of code. This is exacerbated by good programming practices such as creating utility functions for recurrent code and use of domain-specific agent hierarchies (i.e., a specific cell is a subclass of mobile cell, which is a subclass of cell).
- The platform code tangles [24] visualisation, data gathering, and computation with agent encodings, meaning that computation cannot be easily modified separately.
- Lack of clarity on how experimental activities are supported in the code. The documentation describes experimental design in detail; experiments can be re-run exactly; but encoding new experiments or parameterisation requires deep understanding of the code.

In short, there is no efficient way to query the domain modelling, the simulation or experiment design, the rationale, or the code base: to find the answer to even a simple question, the enquirer needs to read all the documentation and understand diagrams and code for the simulator and experimentation.

## Overview of vision

**Fig. 2:**
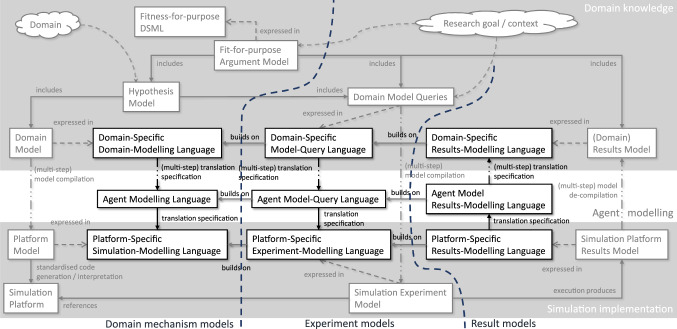
Overview of the family of DSMLs. Outer rectangles relate to existing CoSMoS products and techniques. The inner 3x3 structure is the proposed DSML family. Reading top-to-bottom, it moves from domain-focused to simulation-focused, via software engineering modelling. Reading left-to-right, the focus moves from designing, to querying of designs, to results

Our vision of a *family of DSMLs* to support simulation engineering, Fig. 2, would enable incremental transformation, addressing many of the automation challenges.

Our DSML family centres on a fitness-for-purpose DSML that will allow fitness-for-purpose arguments to be captured explicitly and in an analysable form (cf. Fig. 2, top two rectangles). We believe that the demonstration of fitness for purpose is the key to trustworthy and scientifically robust computational modelling. Fitness-for-purpose arguments demonstrate that the domain has been modelled appropriately for the stated purpose (justifying modelling decisions based on scientific literature, real-world experiments, *etc.*), that the model adequately reflects reality (by showing that it can reproduce results seen in real-world data), and that the simulation experiments are appropriate to establish conclusions (including, but not limited to, showing that the results establish hypotheses with appropriate statistical rigour). The fitness-for-purpose argument must include explicit hypotheses (*e.g.,* about expected behaviours) and explicit modelling of the simulation experiments to exercise the hypotheses.

Fitness-for-purpose analysis cannot be fully automated. However, an explicit fitness-for-purpose model can maintain links between inputs to, and steps of, a fitness-for-purpose argument, enabling systematic inspection, including by researchers outside the study team. We propose to adapt the existing Goal Structuring Notation (GSN) definition by including explicit,^3^ computer-processable links to the other DSML-supported models (and specific versions of these models) that support development and use of the simulation.

The suite of models (diagrams, text, *etc.*) expressing the relevant abstractions of the domain need to be expressed in a domain-modelling DSML so they are accessible to the domain expert. This DSML will likely be adapted to each new research context, to allow natural expression of the domain’s specific concepts. However, we anticipate considerable reuse, supported by modular language components—for example, in cellular biology, there are recurrent mechanisms such as gene-regulation networks or energy minimisation principles, whilst cell clustering and cell differentiation have significant generic aspects. A modular approach to language specialisation also makes it easier to extend models and languages.

To further facilitate reuse and extension, we propose to develop the domain-modelling DSML atop a generic agent modelling language providing the basic concepts for agent-based simulation. The generic language will then be specialised for each specific simulation platform (*e.g.,* [28, 35]), enabling automated transformations of generic agent models into platform-specific simulation models.^4^ We envisage that simulation experiments are also designed and modelled via a hierarchy of languages, with a model querying DSML at the highest abstraction level. Again, such a language has to be specific to each domain, with the potential for reuse across domains, and the use of templates for recurring query types. The query DSMLs should support generation of experiment execution scripts (simulation experiment models) in a stepwise fashion. Explicitly separating queries (in Domain Model Queries) from domain mechanisms (in the Domain Model) is important as it helps avoid encoding the expected emergent behaviours into the simulation. In CoSMoS, this is realised by explicitly separating the Basic Domain Model [44, pp. 130] (containing the hypothesised mechanisms, similar to our Domain Model) from Domain Behaviours [44, pp. 135] (a model of the observed emergent behaviours, similar our Domain Model Queries used from our Hypothesis Model). The latter are explicitly ignored when constructing a Platform Model to avoid tainting the simulation implementation with knowledge about the expected emergent behaviours.

Once simulation experiments have been run, the results need to be presented to domain experts for interpretation. This requires translation back from a platform results model (*e.g.,* a log file of a simulation run) to a domain-specific results model expressing results in terms of the domain queries and domain-model concepts, including information about statistical significance. Again, this is a stepwise translation via an intermediary agent-based results model. Results models are referenced from fitness-for-purpose argument models, ensuring the full end-to-end argument is documented. Thus, fitness-for-purpose arguments become live models tracking the current simulation rationale.

### Expected benefits

Automated generation of executable simulations (Simulation platforms in CoSMoS and Fig. 2) enables *separation of concerns:* domain experts can focus on expressing their mental model of the domain, whilst software engineers can focus on simulation implementation. Further benefits arising from our vision of a family of DSMLs include the following:

- Automated generation means simulations consistently implement the domain model using well-defined transformations that can be inspected by domain experts and software engineers when maintaining the fitness-for-purpose argument. Because fitness-for-purpose is modelled explicitly, the specific implementation of the transformation can be directly referenced from a fitness-for-purpose argument, enabling complete traceability. This is impossible where simulations are manually developed from domain models.
- In generating simulation experiment models, the automated generator can take into consideration expected boundaries for statistical significance and choose appropriate sensitivity analyses for robustness checking—for example by building on tools such as $\hbox {MC}^2\hbox {MABS}$ [20] or Spartan [5]. Again, the generation rules are explicit artefacts that can be referenced from the fitness-for-purpose argument and inspected as needed.
- Building a hierarchy of DSMLs with stepwise translation improves reuse: languages closer to the simulation platform are more likely to be reusable for different types of simulations in different domains. Simulation has been studied for a long time, so simulation platforms are largely stable and do not change substantially. Equally, many domain problems can be simulated using the same fundamental agent-based concepts, but there are problems that will require different concepts to be efficiently simulated. While some aspects of domain models may be reusable across different specific domains (*e.g.,* gene regulation network models), potentially allowing libraries of reusable model components to be created, many aspects of domain models may require highly domain-specific languages.
- Stepwise transformation in a hierarchy of DSMLs also simplifies inspecting the transformation specification by domain experts (together with software engineers). Analysing individual transformation steps induces lower cognitive load. Analyses of lower-level transformation steps can be reused; these do not have to be re-inspected every time. Thus, a hierarchical argument for fitness-for-purpose can be constructed, increasing acceptance and trust.

## User scenarios

We have identified four user scenarios where we expect a model-driven approach to simulation development as outlined in Sect. 3 to be beneficial:

1. *Tracking of fitness-for-purpose artefacts*. As the team’s understanding of the domain and the problem under study develops, so do the various models being developed, the simulations being run and the experiments these enable, the results obtained, and the overall argument for fitness for purpose. Any set of results needs to be mappable to the specific models and simulator code, and the specific fitness arguments. For instance, when it comes to writing a paper, the paper authors need to identify which (versions of the) artefacts contribute to the overall scientific argument and how they do so. But scientific work does not stop with one paper. The simulation and models are likely to evolve and be extended to address new challenges and hypotheses. At any point, it is important to track existing fitness arguments efficiently, and to verify whether extended and refined models still adequately support the original results, identifying where changes have emerged.
2. *Explicit domain translation*. A key step in the development of a computational simulation is the translation of a domain model into a platform model and executable simulation [44, pp. 149–152]. Typically, this is a manual translation process. While CoSMoS patterns such as Seamless Development [44, pp. 214f] aim to make the translation as direct as possible, a translation is invariably required as simulation development moves from the language of the domain to the language of formal computation. This creates problems: it becomes difficult to validate the fitness of a given simulation platform as it is difficult to link implementation choices back to domain concepts, reducing reproducibility and maintainability as well as making it difficult to bring new team members onboard. CoSMoS creates significant documentation requirements (e.g., Document Assumptions [44, pp. 108f]) to document the relationship between domain concepts and computational implementation. However, documentation that is separate to the implementation is likely to become outdated very quickly.
3. *Statistical analysis*. A key aspect of any experiment is robust statistical analysis. This requires expertise significantly different from the domain expertise required to describe observations and hypotheses. Statistical arguments need to be incorporated into the fitness-for-purpose argument to capture, *inter alia,* whether a sufficient number of runs of a simulation experiment have been executed to control for the aleatory uncertainty inherent to any randomised process (such as a typical computer simulation), or how sensitive the results are to changes in parameter values (identifying key parameters and parameter ranges with largely unchanged simulation outcomes) [3]. This often requires input from experts with training in mathematical statistics.
4. *Experimental design*. Designing a (computational) experiment is an incremental and iterative process requiring the creation and execution of many simulation experiments whose results can be compared to results of laboratory (or other scientific) experiments—for example during simulation calibration—and eventually can be interpreted into insights in the real world, perhaps tested further in new laboratory experiments. Experimental design may require specialisation of any part of the simulation model—identifying the questions or hypothesis to be investigated, determining the data to be collected, changing the mechanisms implemented in the simulator, etc. Each set of experiments needs to be matched to a specific version of models and code, and to specific parameter sets, making the correct and consistent execution of simulation experiments a challenging task.

## Application to example

In this section, we discuss the user scenarios in some more detail, relating them to the example from Sect. 2. We begin by giving some more details about the PPSim example before discussing each user scenario in turn.

### Some more detail on PPSim

We illustrate how our vision contributes to these user scenarios using artefacts from the PPSim simulator development [1, 2]. It is worth noting that, while some of these artefacts are diagrammatic models, they do not originally use any formally defined DSMLs. We will show the original artefacts and then show prototypical reimplementations of part of the artefacts to demonstrate the benefits DSMLs and a model-driven approach would offer.

**Fig. 3:**
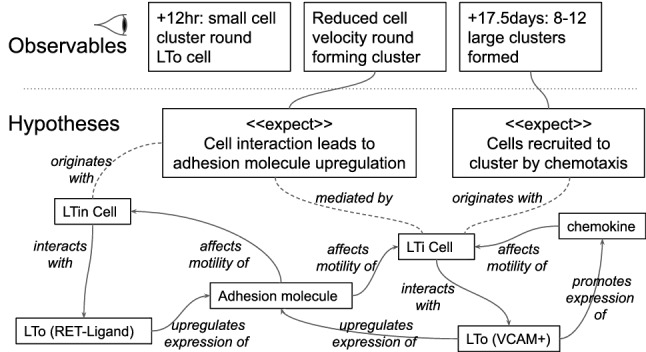
PPSim expected behaviours [1], using a notation devised by York Computational Immunology Labs. “Real world” observables of interest are listed at the top. Those on which the focus rests are elaborated with hypotheses, and each hypothesis is linked to abstract representations of the key “real world” concepts implicated in the hypotheses. These models, which are the result of many hours of collaborative work between domain experts and developers, are used to derive a simulation purpose and the level of abstraction for the simulation development

It is useful, at this point, to give some more detail of the PPSim simulation activity. In the original PPSim development, the domain understanding was captured diagrammatically—an example from [1] can be seen in Fig. 3.

The agreed simulation purpose focused on an exploration of the mechanisms and actors in the formation of Peyer’s patches—clusters of cells that are ultimately part of the immune system, as formed in a neonatal mouse gut. Experimentation using the simulator established unexpected responses to chemicals that were subsequently tested in vivo. A key part of the simulation activity was to simulate the chemical stimuli and cell behaviours implicated in how cells bind together to form these clusters.

It is useful to give an intuition for what (simulated) binding and clustering entails. The basic context is that cells, of various sizes and properties, move through a tube (representing part of the gut), and may interact both with the surface of the tube and with other cells. Interactions are typically mediated by the state of the environment in the tube, modelling dispersing chemicals, flowing fluids, etc. Cell *binding* occurs when two cells—or a cell and the tube wall—are determined to have made contact: in reality, cells have receptors on their surface, and chemical bonds are created or broken. Interactions lead to changes of state in the cells: the new state may, for instance, cause one or both cells to emit a chemical, or respond differently to a chemical, or to differentiate to a state that enables different capabilities. Some of the cell states are labelled (biologically and in simulation) as “bound”, and it is the persistence of a group of bound cells that forms a cluster. Binding is not a once-and-forever action; the bind may be strengthened or weakened by interactions with and around the bound cells.^5^ Note that here, as in much of the natural world, terms such as “bound” and “cluster” are labels applied by scientists to what they observe: within the context and purpose of the simulation, there is no exact definition of when a cell is bound or a group of cells becomes a cluster.

In PPSim, the simulation is designed to cover a period of a few (specific) days in the development of an embryonic mouse gut. Unlike the real mouse “system”, the simulation of the behaviours starts with one type of cell already bound to the wall of the gut (the behaviours preceding this state are not relevant to the simulation purpose). The simulation ends at the point in simulated time that corresponds to the observed real-world appearance of significant cell clusters. Two similar but distinct types of cell are simulated moving through the tube/gut, with appropriate simulated behaviours, depending on the current state of each of the cells. The simulator design is thus a set of state changes, which model the known biological interactions that result in cell binding, including chemical expression and attraction (chemotaxis) by cells. Clusters can be identified visually (as they are in real-world experiments), but cell states include a variable that indicates whether a specific cell is bound or not, and a cell behaviour can probabilistically break a bind, mediated by the simulated chemical environment. Binding has potential self-reinforcement through both chemicals emitted by bound cells, and by further contacts from cells attracted by chemotaxis on such chemicals.

Whilst ongoing binding and recruitment of further cells is aspecific, the first bind relevant to the formation of a cluster is critical: it takes place between a mobile cell and a cell that is already bound to the tube/gut wall (i.e. the cells that exist in the initial state of the simulation). Both cells must be in the right state to take the first step towards binding once the initial, ephemeral, contact is made.

The PPSim simulation goal is simply to model the behaviours from which clustering of cells emerges, over a short period of time. This strict purpose and focus makes the simulation computationally feasible by limiting the range of behaviours, cell states, environmental chemicals, and so on, that need to be simulated. In reality, beyond the simulated time-window, the cluster itself eventually assumes identity and behaviours (becomes a Peyer’s patch), but this is out of scope for this simulation.

Again, it is worth noting that the point at which bound cells become a cluster is not well defined. A scientist can examine a section of gut and count the clusters, identifying each intuitively, perhaps based on their understanding of the subsequent phases of development where a cluster assumes the form and behaviour of a Peyer’s patch. In the simulation, cell states include variables that record contact and binding, to enable appropriate probabilistic behaviours. Since all clusters start from a single cell bound to the cell wall (of a type different to the mobile cells), the simulator can “count clusters” by setting a threshold value for the number of cells that are in contact and bound to the originating cell which is part of the initial state of the simulator. Terms like stable binding can be defined in biological terms, but assume a different sort of meaning computationally.

Next, we discuss each user scenario in turn.

**Fig. 4:**
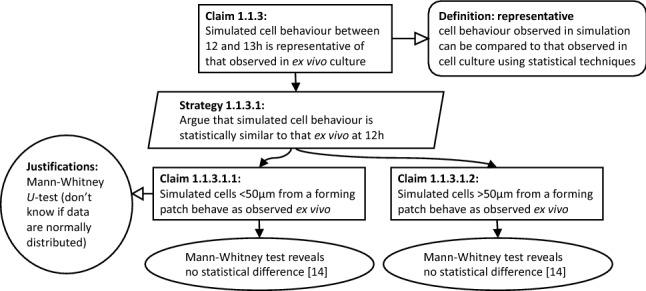
Extract of argument that the PPSim simulation is an appropriate representation of the biological domain for this simulation project, using the CoSMoS variant of Goal Structuring Notation [4, 44]

### (US1) tracking of fitness-for- purpose artefacts

We begin by discussing user scenario (US1), concerning the creation and maintenance of fitness-for-purpose arguments. The rationale for the PPSim model can be expressed in argument diagrams syntactically based on GSN (cf. Fig. 4). However, these models are essentially structured text, providing no explicit link to any of the concrete artefacts comprising the overall simulation model. The argument in Fig. 4 is only valid for a particular version of the simulation model and, in fact, relies on the results from a particular set of runs of the simulation. It also references data from relevant scientific publications of wetlab experiments (“[14]” in the figure), but this link is not explicitly encoded and quickly becomes difficult to track. It is possible to use hyperlinks within GSN, rather than text references such as “[14]” that have no inherent meaning, but this does not guarantee traceability over time.

Similarly, the Peyer’s-patch domain can be represented using a diagram like the one shown in Fig. 3. To date, this form of diagram does not have a formalised notation, but it could be presented in a DSML. The “Observables” at the top of the diagram correspond to domain model queries in Fig. 2 while the “Hypotheses” below correspond to the domain model capturing the hypothesised and to-be-simulated mechanisms.

**Fig. 5:**
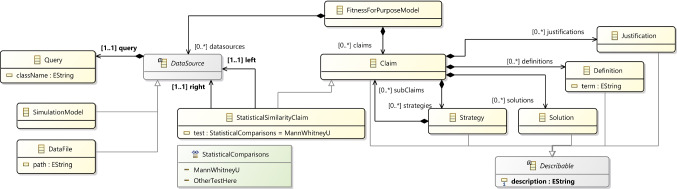
A first meta-model for capturing fitness-for-purpose arguments that link to simulation model artefacts

Building on these observations, Fig. 5 shows an initial meta-model for a fitness-for-purpose DSML. The right part shows the standard GSN concepts of claims, justifications, definitions, solutions, and strategies, each allowing for a textual description.^6^

The Domain-Specific Domain Modelling Language for PPSim would define a Simulation Model concept that represents a container for all domain-model concepts. A specific instance of this concept can be referenced directly from the fitness-for-purpose argument model (see the Simulation Model concept in Fig. 5). Similarly, the Domain-Specific Model-Query Language for PPSim would, *inter alia*, define a concept of Query, with any specific observable being an instance of this concept—also making this explicitly referencable from the fitness-for-purpose argument model.

As we can see, a fitness-for-purpose argument model formalised on the basis of the meta-models above explicitly references all the specific artefacts making up the computational model. This enables the use of standard software versioning tools, such as Git [8], for tracking consistent sets of artefacts—and thus complete fitness-for-purpose arguments—as models and simulations change incrementally and iteratively. Such tracking can be done in different ways:

1. A simple set up would place the fitness-for-purpose argument model into the same repository as the various artefacts it references. This way, every version checked out from the repository contains a specific version of the fitness-for-purpose argument together with the *corresponding* versions of the artefacts it references. This works well for iterative development of one fitness-for-purpose argument, including maintaining a record of model versions underpinning specific scientific publications—the corresponding versions can be tagged using the relevant mechanism of the versioning software used.
2. Where there is a need to develop different experiments on top of a shared (and possibly iteratively refined) domain model, this simple set up may not be sufficient: changing one fitness-for-purpose argument may require changes to some of the underlying artefacts, but these changes may break the validity of other fitness-for-purpose arguments. Keeping all fitness-for-purpose argument models in the same repository together with the underlying artefacts, while technically possible, makes it impossible to track the specific artefact versions attached to specific fitness-for-purpose arguments. The core problem is that fitness-for-purpose arguments and the underlying artefacts may evolve on different time scales. This can, however, be supported by modern versioning systems. For example, in the context of the Git versioning system, one solution might be to place each fitness-for-purpose argument into its own repository referencing the repository/ies containing the underlying artefacts through Git submodules.^7^ In this way, fitness-for-purpose models and the underlying artefacts can evolve independently while still ensuring that for each fitness-for-purpose argument version it is always explicitly clear which precise version of each artefact it references.

Combined with support for citable code (*e.g.,* the GitHub–Zenodo integration^8^), this completely supports user scenario (US1).

### (US2) explicit domain translation

In the PPSim development, Fig. 3 summarises extensive dialogue between scientists and simulation developers, and expresses the abstraction level and potentially-simulatable scenarios, based on observations (data, visualisable emergent behaviours/structures) from real-world experimentation. The next phase of PPSim development, leading to the platform development, was to deduce behaviour diagrams. The simulator code was deduced from the diagrammatic design (and a lot of supporting text) [1]. Focusing on the step from state machine diagrams to code, Polack [38] explores the undocumented manual derivation, and how this might be replaced by transformation, noting the challenges of tracability and documentation of a manual complex system simulation development. This work also identified the way in which some of the non-diagrammatic information was used in manual design and coding, and showed how a systematic translation of the design resulted in often-subtle differences in the derived code structure.

In our vision, we need to establish how these manual derivation can become a series of model transformations. To improve the reproducibility of the simulation experiments and enable a clearer—and maintainable—argument for fitness for purpose, it would be beneficial to capture the translation as a model transformation between the domain model and the simulation platform. This would first require formalisation of the key domain concepts (the Glossary [44, pp. 127f]) as a meta-model so that the transformation into an agent model and, eventually, a platform model can be specified using an existing transformation language. In defining the transformation specification, the need for additional information may become evident; just as additional information became relevant when manually generating the simulation implementation before. Different to the manual approach, this would trigger an incremental extension of the domain-modelling meta-model, ensuring that all relevant information is explicitly captured in a structured artefact.

A similar effect occurs in relation to the “Observables”—Fig. 3—remember that these would be captured in the domain-specific query modelling language. For example, consider the observable ‘small cell clusters around LTo cell’. To express this in a formal model, we need to be able to have concepts such as ‘cell cluster’ or ‘LTo cell’ in our domain-modelling language. An agreed (possibly parameterised) definition of what it means for cells to form a cluster, and the rationale for this, could also be defined, with appropriate transformations into computation, so that a change in the agreed cluster definition could be easily translated into a change in the simulator platform code. By focusing attention away from manual traceability issues, this would allow more effective consideration of any side-effects of the change in definition (e.g. required changes in visualisation or experimental design).

In the PPSim example, binding of cells is captured *statically* via a reference between objects representing cells and *dynamically* via a set of operations that encode the conditions under which cells bind or unbind. Having an explicitly specified transformation between the concept of ‘cell cluster’ and these computation-oriented concepts makes the transformation explicitly inspectable and subject to discussion as well as referencable from a fitness-for-purpose argument. Our proposed transformation approach, linking models, queries over models, and fitness for purpose, would enable, for instance, differentiation in the simulator between “small” and “large” clusters, or between cell-type composition of clusters, without manual adjustment of the codebase. We could then generalise from this simulation instance, for instance to provide a more general expression language as part of the domain-specific model-query language to allow domain experts to capture different classifications of cell clusters that interest them, enabling new simulation experimentation and hypothesis creation without manual reworking of the simulator.

### (US3) statistical analysis

Another benefit of the model-based approach is that it enables separation of concerns and contribution of expertise from different backgrounds. In the example, assessing whether simulation results are aligned with real-world experimental data requires an appropriate domain model, but also requires significant statistical expertise to ensure the simulation results are interpreted correctly. Authors of fitness-for-purpose arguments may not have expertise in all of these areas. However, the model-driven approach means that some expertise that is required across domains can be encoded into the DSML. This is related to the notion of generic arguments proposed in [44, pp. 200ff] to capture recurring argument structures in simulation engineering. An MDE-based approach can go one step further by (partially) automating the evaluation of these argument structures.

As an illustrative example, the left part of the meta-model in Fig. 5 shows how a particular type of claim can be defined, which would capture an assertion of a statistically significant similarity between data from two separate sources (labelled left and right in the figure). Such a statistical similarity claim would still come with a textual explanation, but would further include explicit, computer-processable information about the kind of statistical test employed as well as the specific data sources compared. A data source has two components: the actual source of the data (in the meta-model this can be a CSV data file—for example, taken from a wetlab experiment or a published paper—or a simulation model) and a query that extracts the specific information required from the data source. We are using the Query concept from the model-query language, so such a query could be one of the observables from Fig. 3.^9^ A Simulation Model data source references a specific domain model and indicates that the data to be compared against comes from a simulation of that domain model.

**Fig. 6:**
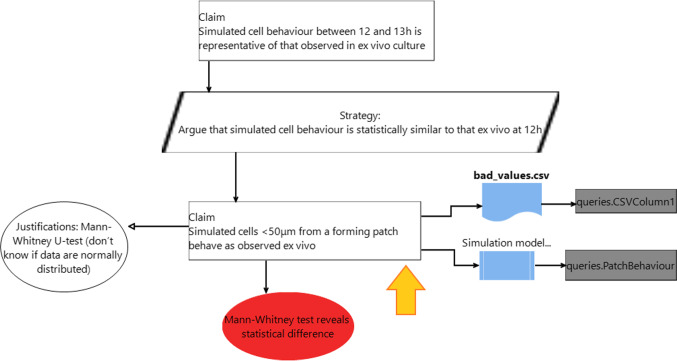
Part of the argument in Fig. 4 modelled in an explicit DSML. Note the red solution shape at the bottom, indicating that the Mann–Whitney U test did not actually confirm that the two sets of data are from the same distribution. The orange arrow is not part of the diagram, but highlights the claim discussed in the text

**Fig. 7:**
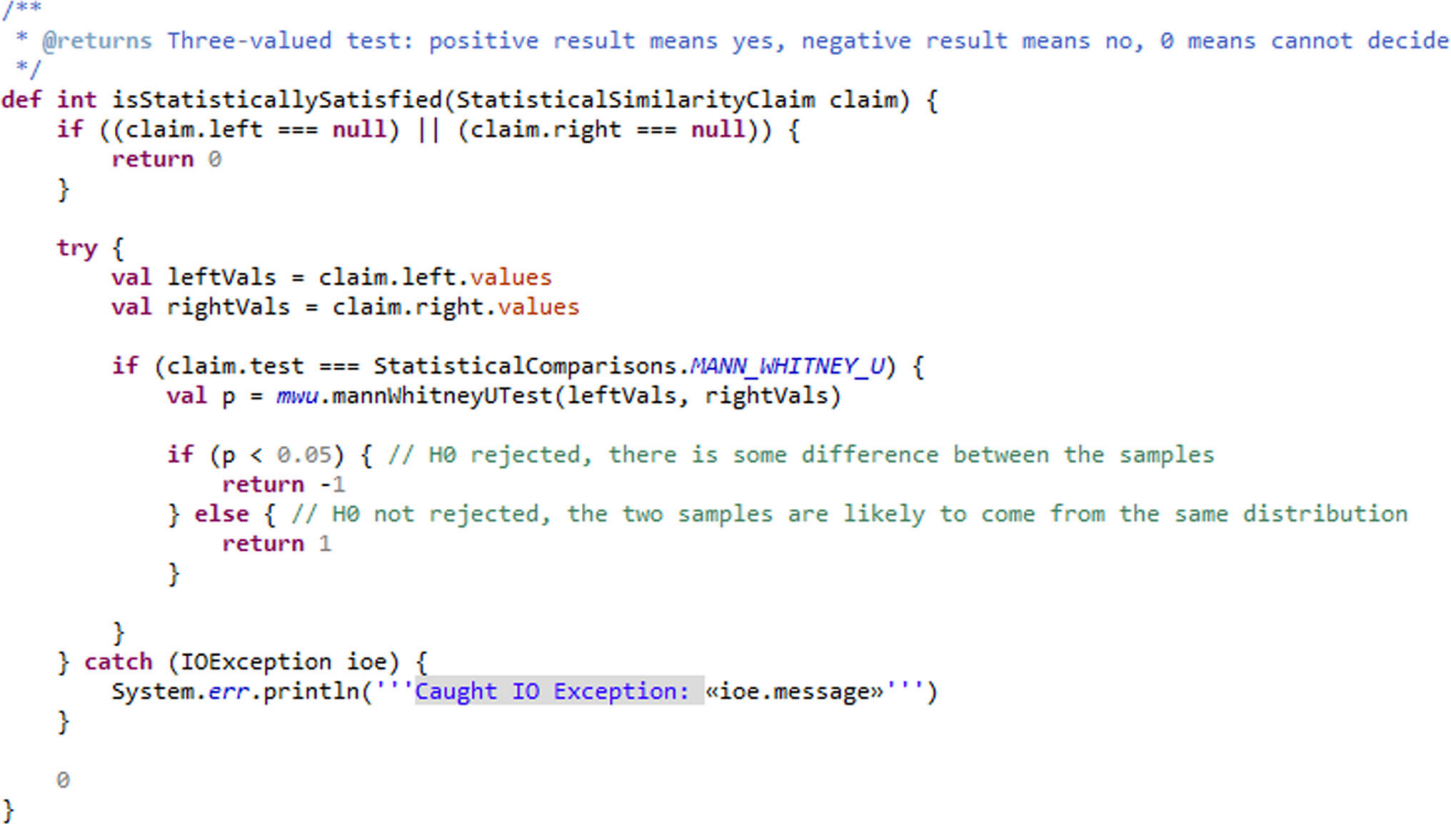
Checking statistical significance of a comparison based on model information

Figure 6 shows the output of a prototype diagrammatic editor that supports generation of and reasoning about the fitness argument for part of the simulation. The editor is built using Eclipse Sirius [46], based on the meta-model from Fig. 5. The diagram shows a subset of the argument from Fig. 4, focusing on part of the statistical claims. The claim towards the bottom of the diagram (indicated by the orange arrow) is an instance of StatisticalSimilarityClaim from the meta-model. It references two data sources: a CSV file taken from a wetlab experiment, from which we extract the data in the first column, and a simulation model, from which we extract information about the patch behaviour. Here, neither the justification on the left, nor the solution entity at the bottom was specified by the user of the tool: the editor added both automatically as soon as the user inserted the claim that the statistical evaluation uses a Mann–Whitney test. The editor generates the argument, and tests it against the identified data sources, using a Mann–Whitney test (cf. Fig. 7). In this illustration, the claim is found not to hold, and the editor thus coloured the offending solution in red to highlight where the fitness-for-purpose argument breaks. This linkage of the argument and the experiment at the DSML level means that the argument, the data, or the model can be changed, and the argument updated automatically: for instance, a different data set (either the real world or simulation data) can be provided, and the fitness for purpose claim re-evaluated. Combined with standard software version-control mechanisms, this approach can satisfy the requirements of user scenario (US1).

### (US4) experimental design

The fitness-for-purpose argument in Fig. 6 simply references the simulation model, but does not specify anything about how this model is to be executed. Knowledge about how to execute a simulation is already encoded in the translation process that takes a domain model and produces a simulation implementation. The execution scripts thus generated can also include statistical analysis—for example using the Spartan tool [3] and its aleatory analysis to determine the number of simulation runs required to allow robust conclusions to be drawn.

The fitness validation shown in Fig. 6 requires running simulation experiments, which can be an expensive task. Validation of fitness-for-purpose argument models, therefore, needs to be triggered by the user. This also allows the experiment executions to be automatically scheduled so as to minimise the computational impact, similar to how modern software build tools automatically schedule the most efficient execution of only required build tasks. All of this execution infrastructure is provided as explicit code and transformation specifications, all of which are explicitly referenced from the fitness-for-purpose argument model, enabling full traceability.

## Related work

Effective development of agent-based models and simulations has been studied for some time, and model-driven approaches have been explored. As a result, some pieces of our vision have already been studied in various contexts. *However, to the best of our knowledge, a vision addressing the entire argument an agent-based model and simulation supports, from the original hypotheses, to the simulation implementation, the simulation experiments, the calibration and model validation and to the final conclusions, has yet to be achieved.* In this section, we point out existing work that addresses some parts of the vision. We structure the discussion according to the three columns in Fig. 2 plus a section on work around fitness-for-purpose arguments.

### Domain modelling

So far, model-driven approaches to describing the domain under study have primarily focused on developing agent languages and (semi-)automated transformations into platform models. For example, the INGENIAS project [13] introduces an agent-modelling language to support replication by enabling the automated translation to different simulation platforms. MAIA [16] is a similar, slightly more recent approach. Here, there is some support for modelling variables of interest and extracting visualisations from the simulation logs. However, this is not connected to hypotheses, simulation experiments, or rationale for developing the simulation in the first place.

The OCOPOMO project [42] is, to the best of our knowledge, the first approach that partially addresses the need for achieving traceability from the original domain understanding and research context to the simulation implementation and final data, by allowing the inclusion of hyperlinks to the original data. However, models remain at the agent-language level, thus requiring a mental shift for domain experts to understand the models and relate them to their domain expertise. Other examples of model-driven approaches to agent-based modelling and simulation can be found in  [21] (possibly the first such approach) and [41], a more recent approach that provides a more domain-specific visual syntax for its agent language.

In [37], Parunak shows an interesting example of a domain-specific modelling approach for an agent-based model in the defence domain. Although this does not explicitly use language-engineering and model-driven engineering technologies, it is perhaps the closest work in intention and conceptualisation to our vision: high-level domain-specific languages (encoded in a variety of technologies, including spreadsheets) are made available to domain experts to express their domain conceptualisation and are automatically translated into an agent-based model that is automatically simulated and results extracted. While Parunak provides examples of domain-modelling DSMLs, there is no support for explicitly capturing a fitness-for-purpose argument over the agent-based model.

Muñoz et al. [34] describe the use of UML and some bespoke modelling languages for capturing the structure and behaviour in an agent-based model of autonomically driving vehicles and pedestrians, developed for purposes of training and testing autonomic-driving AI systems. This work proposes some domain-modelling DSMLs—for example, the authors provide a DSML for specifying different levels of uncertainty of agents about road conditions and other agents based on levels of visibility, etc. At the same time, however, the approach requires some very low-level ABM features to be modelled, too. In particular, the user is required to produce a UML model that captures the simulation-implementation classes down to the level of tick() methods. Thus, the approach has aspects that are more low-level than the agent-modelling approaches in [13, 16, 21, 41, 42] mixed with approaches that are at a similar abstraction level to the languages introduced in [37] and proposed in our vision. Explicit models of fitness-for-purpose arguments are again not considered in [34].

Barat *et al.* [7] describe OrgML, a domain-specific modelling language for describing organisational structures, levers and outcomes and translating these into agent-based models and simulations. This is similar to the left column in Fig. 2 in that OrgML is a domain-specific domain-modelling language and the underlying actor language is an agent modelling language.

### Query and experiment modelling

In the wider simulation community, it has been recognised that the definition of simulation experiments follows similar patterns across domains and can be captured through a domain-specific language. SESSL [12] is one such language, which allows the specification of simulation experiments and the processing of results—for example for visualisation—in an internal DSL embedded in Scala. SESSL focuses, in particular, on providing a unifying mechanism for specifying simulation experiments that can be executed on different simulation platforms. However, the specification remains at what in our vision we refer to as the platform-specific level: domain-specific or agent-specific concepts are not used in the DSL.

ESS [30] is an external DSL for the specification of simulation experiments. Here, the focus is less on supporting different simulation ‘back ends’ and more on the support for different statistical analyses. However, as with SESSL, ESS also remains at what we would refer to as the platform-specific level and does not provide support to domain stakeholders.

There has been extensive research interest in the validation of agent-based models, and some of this work has started to explore approaches for ensuring the statistical soundness of the conclusions drawn from simulations. For example, Spartan [5] is a toolkit supporting statistical analysis of simulation runs to alleviate aleatory uncertainty and undertake sensitivity analysis, focusing on numerical output data. Similarly, $\hbox {MC}^2\hbox {MABS}$ [20] provides support for drawing statistically sound conclusions based on temporal-logic queries about patterns of agent behaviour. However, neither of these approaches are currently integrated with the initial domain model; they remain at the platform-specific level. As a result, they require extensive manual translation and integration effort from domain experts and software engineers to be applied to a new simulation context.

ProMoBox [31] is an interesting example of a domain-specific query language outside the simulation world. The authors observe that domain experts struggle to work with linear temporal logic expressions and relating them to their domain concepts. To address this challenge, they provide a mechanisms for generating domain-specific query languages by lifting the structure of LTL expressions to the domain level and replacing predicates with patterns in the original domain language. This is achieved via a domain-specific meta-modelling language [53].

### Result modelling

We are not aware of many works on results modelling. Many simulation tools allow the specification of what parameters and attributes should be monitored and exported as results of a simulation run. SESSL [12] discussed in more detail above, includes concepts for describing what data to extract and how to visualise it, in a manner that is independent of specific simulation tools.

### Fitness-for-purpose arguments

Arguing for the validity of experiments and simulations is important and has been studied from different perspectives.

#### Experimental frame and validity frame

As a general concept, Zeigler [51] introduce the “experimental frame” capturing the context of a system under study as the inputs and corresponding outputs allowing to establish a validity argument as a comparison of input–output behaviour of the real system as compared to the simulation system. Denil *et al.* [10] (the foundation for [30]) identify further aspects that need to be captured to support reproducibility of simulation experiments, including information about what data is to be collected and how these observations are made and information about the solvers used. They refer to this extended set of information as the validity frame of a simulation experiment.

In the context of agent-based models in computational science, the CoSMoS approach [44] suggests that a richer set of information is required, linking modelling decisions to the scientific literature, real-world experimentation, expert decision making, etc. They argue that such a rich rationale for the fitness-for-purpose of a given simulation can only be captured as a structured argument, some of which subsumes aspects that are covered by experimental and validity frames. Our vision in Fig. 2 is based on this understanding.

#### Provenance and scientific workflows

One aspect of this is an understanding of how a particular model came to be as a consequence of a series of decisions, linked to previous experiments and scientific papers, and how the execution of an experiment led to the results obtained. A similar argument applies to scientific process descriptions and our envisioned family of languages could form an integral part of a formalised description of an experimental process. An alternative model-based approach to process description is described in [43]. Concepts of provenance have long been applied to the documentation of scientific workflows [32]. Specifically for simulation experiments, the use of provenance has been discussed in [39].

#### Argument structures

We have borrowed the structure of fitness-for-purpose arguments (and the language used for these) from the domain of safety assurance cases. As a result, work in that area is potentially relevant here, too. In particular, we note considerations on pattern/template-based development of assurance cases, on automated instantiation of assurance cases, and on the assurance of DSML-based systems.

Kelly and McDermid [23] introduced the idea of capturing recurring argument structures in assurance cases as *assurance patterns.* Assurance patterns can be considered parametrised partial assurance arguments that are meant to be instantiated into actual assurance cases by linking parameters to data from the system being developed. Yan *et al.* [50] survey different approaches to using assurance patterns for the creation of assurance cases. One of the approaches—also realised in the AdvoCATE tool [11]—constructs assurance cases by, in effect, gluing together instantiated assurance-case patterns. We have previously shown how model patterns (or model fragments) can be abstracted into DSML language constructs [22]. The StatisticalSimilarityClaim concept we introduced in Sect. 5 is an example where we have identified an assurance pattern for part of a fitness-for-purpose argument for simulations and have encoded this as a language concept directly in the fitness-for-purpose DSML. An interesting question for future research is: What are the argument patterns for fitness-for-purpose models in simulation engineering and how can they be best captured as DSML concepts.

Wassyng *et al.* [48] point out that just having a structured approach to describing assurance arguments does not guarantee that *meaningful* arguments will be constructed. They argue for a twofold solution: (1) to use assurance templates (in effect, assurance patterns with additional contextual and guidance documentation for when and how to use them) to drive system development rather than only creating assurance cases as documentation after the fact, and (2) improved structure of assurance-case models that is able to capture not just the top-down decomposition of arguments, but also the logical recomposition of sub-arguments into supporting a larger claim. The latter is also elaborated in [40], which argues for a formalised underpinning of assurance-case models. The use of assurance templates to drive the design and implementation of systems is a very interesting idea; one research question is whether the fitness-for-purpose model can be used to *drive* rather than document the scientific process, perhaps even acting as a form of structured electronic lab notebook [25].

Assurance cases—and we expect the same to be true of fitness-for-purpose arguments—can become quite large and complex themselves. However, some aspects will be largely mechanistic (e.g., connecting to appropriate data sources and establishing relevant properties), while others require more significant human input. In the safety assurance literature, this has been recognised early [14], leading to significant research into (often model-driven) mechanisms for automatically instantiating assurance cases (e.g., [18, 19, 29]). This is typically based on instantiating assurance patterns, explicitly leaving open inputs that need to be provided by humans; offering a way to focus human attention and drive system design based on where the most value can be added to the overall case [48]. An interesting research question in the area of simulation engineering is, then, which aspects of the fitness-for-purpose argument can be instantiated automatically, and which require human construction. [29] also introduces the interesting idea of *defeaters* as part of the assurance case: these “capture doubts and objections” and may be useful in simulation engineering to capture, for example, calibration failures that motivated changes in the model and other information about the incremental and iterative refinement of the model.

In [47], Voelter et al. discuss the risks involved in using DSMLs and the underlying language-workbench technology in safety-critical systems. They provide a catalogue of risks and corresponding technological and process-based mitigations. While they do not provide this, the catalogue would translate quite straightforwardly into an assurance-case template to be instantiated as part of a wider assurance case. The same concerns, in effect, apply to the fitness-for-purpose argument for DSML-based construction of simulations, though the lower criticality of the overall system (in particular, where it is used more for discovery and as the basis for later wet-lab experimentation than for product development) will likely mean that less stringent arguments are required. Nonetheless, the risks and mitigations discussed in [47] should be considered as part of a fitness-for-purpose argument for computational simulations based on DSMLs.

## Conclusions and outlook

We have presented a vision for a family of DSMLs for building robust and trustworthy agent-based simulations, where the models can be understood by domain experts and can be clearly traced to the final simulation and the simulation results, thus constructing an integrated fitness-for-purpose argument. Some parts of the vision have been explored before. However, the combination of modular DSMLs for modelling domain knowledge, model queries and simulation experiments, and computer-analysable fitness-for-purpose arguments has never been explored. We have shown an initial proof of concept for automated argument management, and we are extending our work to prototyping such languages in the domains of computational biology and health improvement science.

In addition to underpinning high-quality, reproducible and maintainable simulation, our vision would allow linkage of simulation with the growing body of work on uncertainty [45]. A range of meta-models now exist for belief uncertainty, partial specification, bounded knowledge, etc. These can be merged with DSMLs to allow arguments that reflect the relative state of knowledge among domain scientists and developers, with a holy grail of establishing the level of trust to be placed on results.

## Footnotes

1 In reality, the link between projects and simulators is not so simple. Some of challenges are illustrated in relation to the revisiting of the PPSim simulation validation in [38]

2 See kennedy.ox.ac.uk

3 https://www.goalstructuringnotation.info

4 Many generic simulation platforms already come with a platform-specific simulation-modelling language, often as a library or framework in a general-purpose language such as Java.

5 A summary of the biology of binding can be found in [4, Fig. 1.4].

6 This is a simplification of the GSN meta-model defined in the GSN standard [6] as a specialisation of the OMG’s Structured Assurance Case Metamodel [36]. For the purposes of this paper, we focus on the key GSN concepts, omitting the more generic SACM concepts.

7 https://www.git-scm.com/book/en/v2/Git-Tools-Submodules

8 https://www.guides.github.com/activities/citable-code/

9 For simplicity, we are encoding queries in Java classes implementing a simple interface. In future work, this would be replaced by a separate DSML for capturing queries of relevance in the domain.
